# DeePMD-GNN: A DeePMD-kit Plugin for External Graph Neural Network Potentials

**DOI:** 10.1021/acs.jcim.4c02441

**Published:** 2025-03-28

**Authors:** Jinzhe Zeng, Timothy J. Giese, Duo Zhang, Han Wang, Darrin M. York

**Affiliations:** † Laboratory for Biomolecular Simulation Research, Institute for Quantitative Biomedicine and Department of Chemistry and Chemical Biology, Rutgers University, Piscataway, New Jersey 08854, United States; ‡ AI for Science Institute, Beijing 100080, P. R. China; § DP Technology, Beijing 100080, P.R. China; ∥ Academy for Advanced Interdisciplinary Studies, 12465 Peking University, Beijing 100871, P.R. China; ⊥ National Key Laboratory of Computational Physics, Institute of Applied Physics and Computational Mathematics, Fenghao East Road 2, Beijing 100094, P.R. China; # HEDPS, CAPT, College of Engineering, 12465 Peking University, Beijing 100871, P.R. China

## Abstract

Machine learning potentials (MLPs) have revolutionized molecular simulation by providing efficient and accurate models for predicting atomic interactions. MLPs continue to advance and have had profound impact in applications that include drug discovery, enzyme catalysis, and materials design. The current landscape of MLP software presents challenges due to the limited interoperability between packages, which can lead to inconsistent benchmarking practices and necessitates separate interfaces with molecular dynamics (MD) software. To address these issues, we present DeePMD-GNN, a plugin for the DeePMD-kit framework that extends its capabilities to support external graph neural network (GNN) potentials.DeePMD-GNN enables the seamless integration of popular GNN-based models, such as NequIP and MACE, within the DeePMD-kit ecosystem. Furthermore, the new software infrastructure allows GNN models to be used within combined quantum mechanical/molecular mechanical (QM/MM) applications using the range corrected ΔMLP formalism.We demonstrate the application of DeePMD-GNN by performing benchmark calculations of NequIP, MACE, and DPA-2 models developed under consistent training conditions to ensure fair comparison.

## Graphical abstract

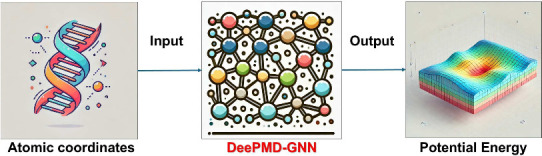

## Introduction

In recent years, many machine learning potentials (MLP) have been developed to model the potential energy of atomistic systems.^1–6^ These developments have resulted in numerous software packages that implement each new MLP;^7–19^ however, the software is often limited to support only those MLPs developed within a particular research team. Some of the popular packages include: DeePMD-kit^7,20,21^ (used to develop Deep Potential models^22–24^ ), SchNetPack^8,16^ (used to develop for SchNet^25^), TorchANI^12^ (used to develop various ANI models^26,27^ ), and the NequIP,^28^ and MACE packages.^29^ The emergence of separate software ecosystems has several disadvantages. First, it is inconvenient and inefficient to have users learn new software with the release of each new MLP. This has led to the release of support software, such as MLatom,^30^ that creates workflows which try to run MLP packages in a unified way. Second, it is inconvenient and inefficient to have developers interface each MLP package with molecular dynamics (MD) software to enable their use in simulation.^14,31,32^ Finally, the different infrastructures make it difficult to train the various models in a consistent manner due to differences in the optimization algorithms, the definition of the loss function, the treatment of learning rates and training steps,^4^ and the availability of active learning strategies.

The present work introduces the DeePMD-GNN package, a DeePMD-kit plugin for external graph neural network potentials. The location of DeePMD-GNN within the broader DeePMD-kit software ecosystem is illustrated in Figure 1. To demonstrate its capabilities, we created plugin interfaces for two popular GNN potentials, NequIP^28^ and MACE.^29^ With the aid of DeePMD-GNN, these models can be trained and used in the DeePMD-kit package in the same way as other Deep Potential models to enable a wealth of applications in chemistry, biology and materials science. Furthermore, the plugin interface allows the GNN potentials to be used within range corrected QM/MM-ΔMLP applications.^33,34^ Semiempirical or approximate density-functional tight-binding methods are computationally efficient, but have inherent limitations^35–37^ that prevent them from achieving the accuracy of much more computationally intensive *ab initio* QM methods. The range corrected QM/MM-ΔMLP strategy uses neural network to introduce short-range nonelectrostatic corrections to an inexpensive (semiempirical) QM/MM base model to reproduce target *ab initio* QM/MM energies and forces. The DeePMD-GNN plugin greatly extends the capability of recently developed interoperable software infrastructure^38,39^ within Amber^40^ for design of next-generation QM/MM-ΔMLP models and their application to biochemical reactions^41,42^ and drug discovery.^43–45^ The new software interfaces are demonstrated by comparing benchmark calculations of NequIP,^28^ MACE,^29^ and DPA-2 ^24^ models developed with a consistent training strategy. The errors are compared using structures from the QDπ data set.^46^

**Figure 1:**
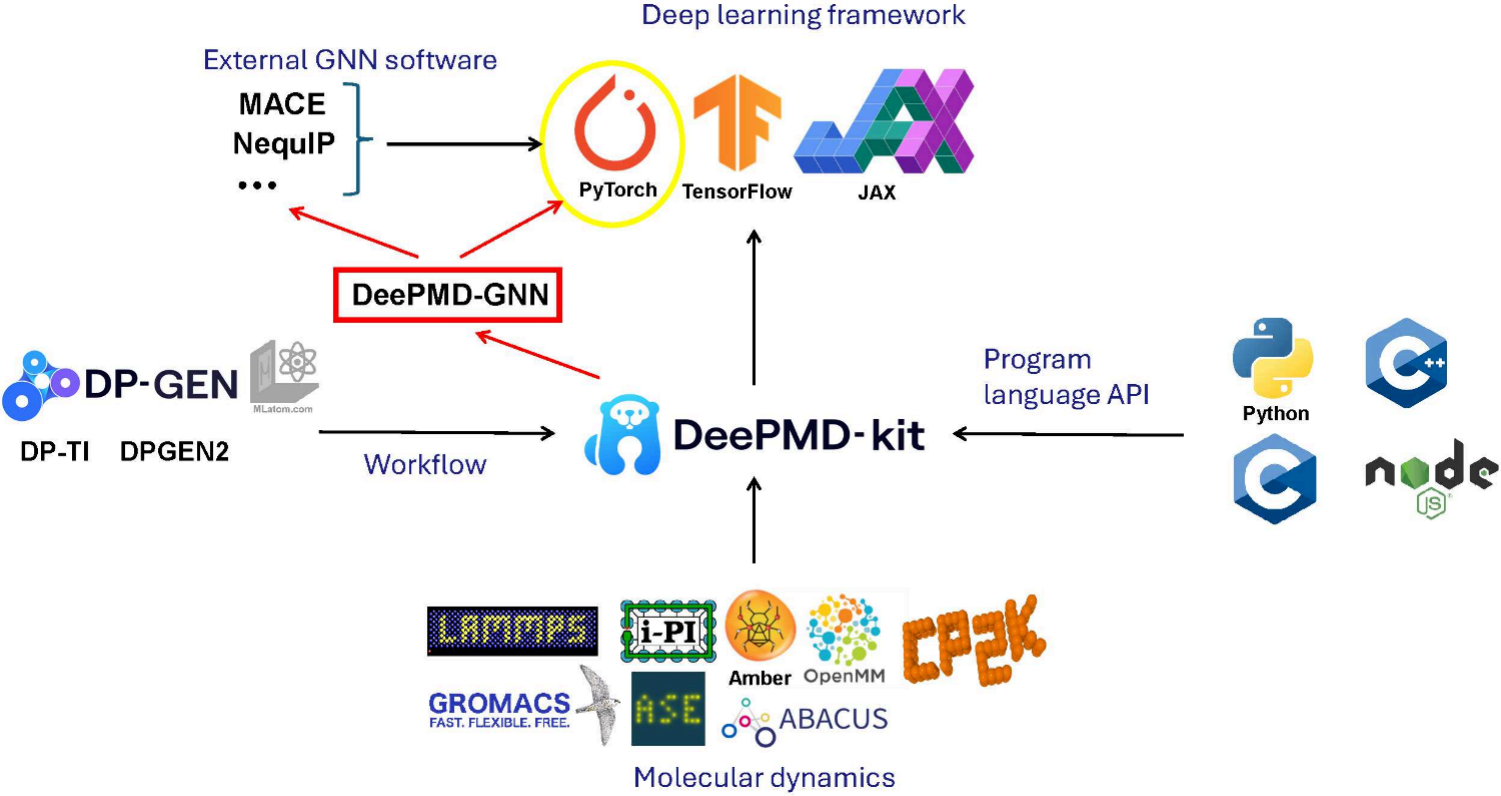
Location of DeePMD-GNN in the DeePMD-kit software ecosystem. The arrows indicate dependency flow, and red color indicates new software and interfaces developed in the current work. Software packages shown in the figure include (1) DeePMD-kit^20^ and DeePMD-GNN, (2) External GNN software: MACE,^29^ NequIP,^28^ and so on, (3) Deep learning framework: TensorFlow,^47^ PyTorch,^48^ and JAX,^49^ (4) Molecular dynamics packages: LAMMPS,^50^ i-PI,^51^ Amber,^40^ OpenMM,^52^ CP2K,^53^ GROMACS,^54^ ASE,^55^ and ABACUS,^56^ (5) Workflow packages: DP-GEN^57^ and its next generation, MLatom,^30^ and DP-TI. (6) Program language API: Python, C, C++, and Node.js.

## Software Description

The DeePMD-GNN package is an open-source project hosted on GitHub and licensed under LGPL-3.0.It is a Python/C++ mixed source project that is packaged with CMake^58^ and scikit-build-core.^59^ The software dependencies include DeePMD-kit,^20^ NequIP,^28^ MACE,^29^ and PyTorch.^48^

### Software Infrastructure

The software infrastructure used to train and apply MLPs is illustrated in Figure 2 to highlight the components provided by the DeePMD-GNN package. The diagram depicts two use cases: model generation by concurrent learning and model inference within molecular simulation applications. The DP-GEN software^57^ provides an interface to the DeePMD-kit Python package^20^ to train a model; that is, optimize the network parameters. The DeePMD-kit Python package is interfaced to the external GNN PyTorch software via a generic model wrapper, and the graph edges are prepared by a custom C++ operator library provided by DeePMD-GNN. When the DeePMD-kit Python package has finished the parametrization, it saves the GNN and its parameters to a serialized TorchScript model file. To use the trained model in a molecular simulation, one must run a version of the MD software that has been interfaced to the DeePMD-kit C/C++ library. The DeePMD-kit C/C++ interface can load and evaluate the saved TorchScript model file with the aid of the C++ operator library provided by DeePMD-GNN. Consequently, it is not necessary to implement the C/C++ interface for each Python-implemented GNN model, thus simplifying the integration process. The DP-GEN software can also train models using a query-by-committee active learning strategy that involves parametrization of several network parameter sets. DP-GEN will then conduct exploration for additional training data by using the current model parameters within MD simulations. If a simulation encounters a sample that produces significant disagreement between the models, then it is saved. A subset of the saved samples are selected at random for labeling and used to parametrize the network in the next active learning iteration.

**Figure 2:**
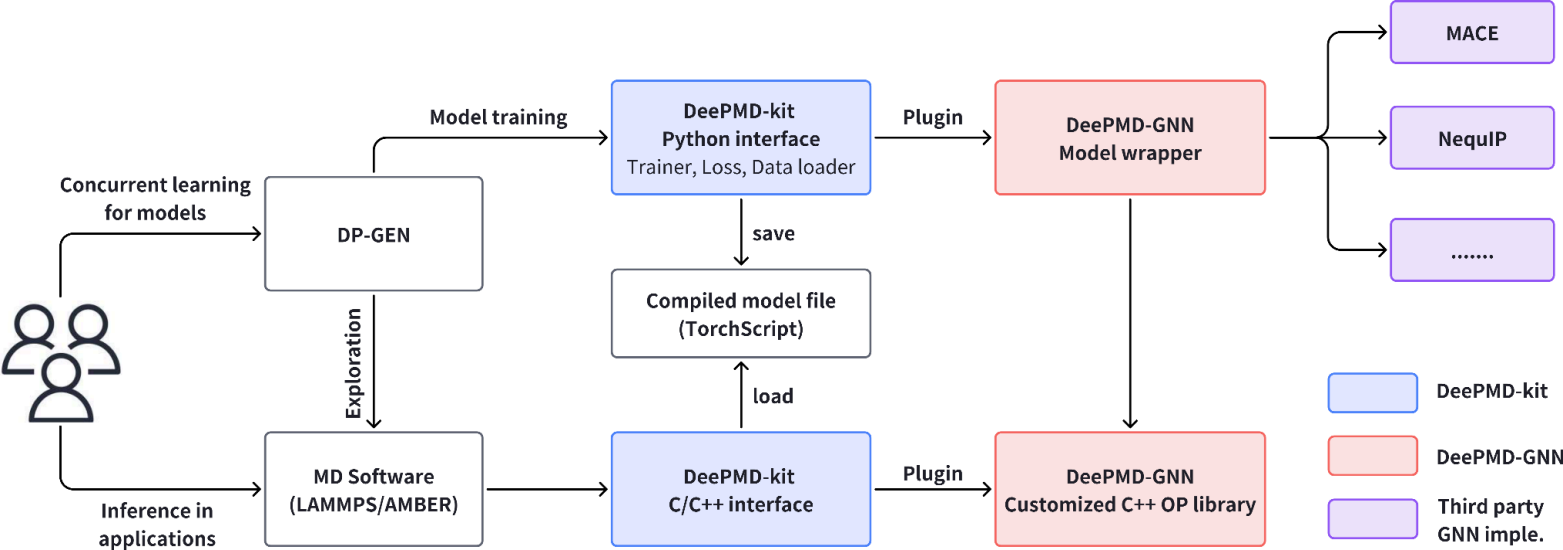
Software architecture of the DeePMD-GNN package. The boxes represent software components, and the arrows represent dependency between the software and the flow of data. A → B means that software component A depends on B; A calls B with input data, and B returns the output back to A.

### Software Features

The DeePMD-GNN package adapts the Deep Potential-Range Correction (DPRc) method^33^ for use with GNN potentials for the development of range-corrected GNN models. This method is used to create ΔMLP corrections for semiempirical quantum models in QM/MM applications,^34,60^ where the total potential energy is the sum of the QM/MM and MLP energies.

1 $$E=E_{QM}+E_{QM/MM}+E_{MM}+\Delta E_{MLP}$$

A range-corrected ΔMLP potential corrects both the QM and the nearby QM/MM interactions in a manner that produces a smooth potential energy surface as MM atoms enter and exit the vicinity of the QM region. To use GNN potentials with this approach, the MM atom energy bias is set to zero and the GNN topology excludes edges connecting pairs of MM atoms. The application and comparison of GNN and Deep Potentials range-corrected ΔMLP QM/MM applications using the DeePMD-GNN infrastructure will be the subject of forthcoming work.

## Benchmark Comparison of Graph Neural Network Models

A key usage of the DeePMD-GNN package is to train and benchmark different GNN potentials in a consistent manner. As a brief demonstration, we present benchmark calculations using the DPA-2,^24^ MACE,^29^ and NequIP^28^ potentials. These GNNs are trained for use as pure MLPs and QM-ΔMLPs, where the ΔMLP is a correction to the GFN2-xTB semiempirical method.^61,62^

The total energy of the QM-ΔMLP model is the sum of the GFN2-xTB and MLP energies.

2 $$E=E_{GFN2-xTB}+\Delta E_{MLP}$$

The target energy Δ*E*_MLP_^*^ to be learned by the ΔMLP model is the difference between the *ab initio* reference and GFN2-xTB methods.

3 $$\Delta E_{MLP}^{*}=E_{ref}-E_{GFN2-xTB}$$

In the present work, the *ab initio* reference method is ωB97M-D3­(BJ)/def2-TZVPPD. Each model is trained consistently against the QDπ data set^46^ which includes energies and forces calculated with ωB97M-D3­(BJ)/def2-TZVPPD^63^ for over 1.5 million structures that were collected from subsets of the SPICE^64^ and ANI,^65,66^ data sets, in addition to smaller data sets that include neutral and charged compounds covering the chemical space of 15 elements: H, Li, C, N, O, F, Na, P, S, Cl, K, Br, and I. The QDπ data set is split into training and test sets with a 19:1 ratio. The DPA-2 model is benchmarked at three different sizes: small (S), medium (M), and large (L). The DPA-2 (S), DPA-2 (M), and DPA-2 (L) models use 3, 6, and 12 representation-transformer (reperformer) layers, respectively. The DPA-2 (M) and DPA-2 (L) model’s reperformer pair-atom representation is updated with a gated self-attention layer, whereas the DPA-2 (S) model is not. The remaining hyperparameters are the same in the model sizes. Specifically, the representation-initializer layer is encoded from the local environment within a 6 Å cutoff radius and 1 Å of smoothing. The reperformer layers are calculated with a 4 Å cutoff and 1 Å of smoothing. Three-body embedding is included within a 4 Å cutoff. The embedding network consists of 3 hidden layers with 25, 50, and 100 neurons. The embedding submatrix size is 12. The fitting network consists of 3 hidden layers with 240 neurons, and the dimensions of the invariant single-atom and pair-atom representations are set to 120 and 32, respectively. Furthermore, the localized single-atom representation update mechanism excludes the self-attention layer.

The MACE model is benchmarked at two different sizes that differ only in the maximum rotational order used to communicate equivariant messages. The MACE (S) model’s message passing mechanism uses a symmetry order of 0 with 256 embedding channels, and the MACE (M) model uses a symmetry order of 1 with 128 embedding channels. The remaining hyperparameters are the same between the two models. The radial features are calculated from a 6 Å cutoff, 8 Bessel functions, and a order 5 polynomial envelope. The features were fed to a 3-layer perceptron consisting of 64 neurons/layer. The angular description of the environment is expanded in spherical harmonics to order 3. The MLP is calculated from 2 message passing layers with a correlation order of 3.

A single NequIP model is trained. The radial features are calculated from a 6 Å cutoff, 8 Bessel functions, and embedded with a 1-layer perceptron consisting of 64 neurons/layer. The MLP consists of 4 message passing layers using a maximum irreducible representation order of 2, and the hidden features were configured to use a maximum order of 1 using 32 channels and both even and odd parity.

All models are trained with the same loss function, learning rate, training steps, and floating point precision (FP32) using the Adam stochastic gradient descent method.^67^ The number of training steps is set to 1 million. The learning rate exponentially decays from 10^–3^ to 3.51 × 10^–8^. The weighted contribution of the energy errors to the loss function increases from 1 eV^–2^ and 20 eV^–2^ during the training, whereas the contributions from the force errors decrease from 100 to 1 eV^–2^Å^2^. The batch size is set to ⌈256/*N*⌉, where *N* is the number of atoms in a conformation. In previous studies, MLP models trained with different random seeds typically yield similar error statistics.^68,69^ This consistency indicates that the randomness inherent in the training process is not significant, and therefore we do not treat the random seed as a parameter in this demonstration.

Table 1 shows the energy and force mean absolute errors (MAE) and root-mean-square errors (RMSE) of the DPA-2, MACE, and NequIP models against the QDπ data set. The GFN2-xTB+ΔMLP models are consistently better than the pure MLP models. This observation is consistent with previous comparisons that used Deep Potential models.^22–24^ Among the pure MLPs, DPA-2 (L) yields the lowest errors, and the NequIP model produces the largest errors.Among the GFN2-xTB+ΔMLP models, the ΔDPA-2 (L) and ΔNequIP models similarly produce the lowest and largest errors, respectively.

**Table 1:** Energy (E, in unit kcal/mol) and Force (F, in unit kcal/(mol·Å)) Mean Absolute Errors (MAE) and Root Mean Square Errors (RMSE) of Several GNN Models against the QD*π* Data Set^a^

	Training set				Test set				
Model	E MAE	E RMSE	F MAE	F RMSE	E MAE	E RMSE	F MAE	F RMSE	*t*(infer)
**Pure MLPs**									
DPA-2 (S)	3.19	7.26	3.12	8.11	3.19	5.18	3.11	5.94	2728
DPA-2 (M)	2.02	6.28	2.04	7.27	2.02	3.65	2.03	4.70	6996
DPA-2 (L)	1.73	6.07	1.77	7.08	1.75	3.32	1.77	4.43	13713
MACE (S)	2.56	7.03	2.25	7.54	2.55	4.73	2.24	5.09	2585
MACE (M)	1.98	6.62	1.74	7.17	1.97	4.09	1.74	4.54	4723
NequIP	4.49	8.88	3.65	8.79	4.46	7.12	3.64	6.80	1622
									
**QM**									
GFN2-xTB	–	–	4.36	9.58	–	–	4.38	7.84	4048
									
**QM-ΔMLPs**									
ΔDPA-2 (S)	1.27	5.70	1.25	6.64	1.27	2.58	1.25	3.82	6776
ΔDPA-2 (M)	0.98	5.57	0.99	6.53	0.98	2.31	0.99	3.63	11044
ΔDPA-2 (L)	0.89	5.54	0.92	6.50	0.89	2.23	0.92	3.58	17761
ΔMACE (S)	1.19	5.71	1.08	6.60	1.19	2.60	1.07	3.73	6633
ΔMACE (M)	0.95	5.61	0.85	6.51	0.95	2.38	0.85	3.57	8771
ΔNequIP	1.75	6.02	1.46	6.79	1.74	3.22	1.45	4.06	5670

a QM-ΔMLP models prefixed by Δ use GFN2-xTB as a base QM model that are supplemented by a ΔMLP correction. Also shown are uncorrected QM models at the semiempirical GFN2-xTB level. *t*(infer) is the inference time (s) for the whole training set. The MLPs were evaluated on a single NVIDIA V100 GPU card, and the GFN2-xTB semiempirical energy was calculated on 32 AMD EPYC 7742 CPU cores.

Table 1 also shows the inference time needed to calculate the whole training set with a single NVIDIA V100 GPU card and 32 AMD EPYC 7742 CPU cores, where the MLP is evaluated on the GPU and GFN2-xTB is calculated on the CPUs. The pure MLP models can be ordered from most to least expensive to evaluate: DPA-2 (L) > DPA-2 (M) > MACE (M) > DPA-2 (S) > MACE (S) > NequIP. The GFN2-xTB+ΔMLP models are about 1.5 times more expensive than the pure MLP models.

## Conclusions

The DeePMD-GNN package makes a significant step forward in addressing key limitations in the current MLP software ecosystem and advancing the state-of-the-art enabling technology for molecular simulations using MLPs. By enabling the integration of external GNN potentials, such as NequIP and MACE, within the DeePMD-kit framework, it reduces the need for users to learn multiple software packages and ensures consistency in benchmarking practices. Furthermore, the DeePMD-GNN package includes infrastructure allowing the DeePMD-kit C/C++ library to read and use GNN models saved as TorchScript files. In this manner, PyTorch implementations of GNN models become immediately available in MD software that is interfaced to DeePMD-kit. The incorporation of the range-corrected ΔMLP strategy within DeePMD-GNN further allows GNN models to be used as corrections for semiempirical QM/MM calculations. We benchmarked several GNNs against the QDπ data set to highlight the utility of DeePMD-GNN in providing a unified platform for fair and efficient evaluation of advanced MLP methods. DeePMD-GNN is also the first plugin developed for the DeePMD-kit package, and thereby serves as an example for future development of plugins. Of particular note is the interface of DeePMD-kit with new software infrastructure in the Amber software package that enables simulations next-generation QM/MM-ΔMLP models together with a wide range of advanced alchemical free energy and free energy surface methods. While our current implementation leverages the two most popular and well-established GNN-based atomistic models, additional models can be easily integrated into the DeePMD-GNN package, ensuring that the DeePMD-GNN package remains at the forefront of methodological advancements. Moreover, as a plugin for DeePMD-kit, our tool benefits from its evolving features and integrations; any enhancements introduced by DeePMD-kit will be seamlessly supported by the plugin without necessitating modifications. The GNN plugin infrastructure assumes that the MLP is a short-range nonelectrostatic correction whose input depends only on the atomic coordinates and elements involved. This limitation would present challenges in the future if new GNN potentials are developed that expand the model inputs to include atomic charges, for example.

We anticipate that DeePMD-GNN will facilitate a wide range of new applications that leverage GNNs to gain predictive insight into drug discovery, biocatalysis and materials design.

## Data Availability

Source code for the project can be found at https://gitlab.com/RutgersLBSR/deepmd-gnn or https://github.com/deepmodeling/deepmd-gnn.
